# Smart Cybersecurity Framework for IoT-Empowered Drones: Machine Learning Perspective

**DOI:** 10.3390/s22072630

**Published:** 2022-03-29

**Authors:** Abdulaziz Aldaej, Tariq Ahamed Ahanger, Mohammed Atiquzzaman, Imdad Ullah, Muhammad Yousufudin

**Affiliations:** 1 College of Computer Engineering and Sciences, Prince Sattam Bin Abdulaziz University, Al-Kharj 16278, Saudi Arabia; t.ahanger@psau.edu.sa (T.A.A.); i.ullah@psau.edu.sa (I.U.); m.yousuf@psau.edu.sa (M.Y.); 2 School of Computer Science, University of Oklahoma, Norman, OK 73019, USA; atiq@ou.edu

**Keywords:** security, machine learning, drones, Internet of Things

## Abstract

Drone advancements have ushered in new trends and possibilities in a variety of sectors, particularly for small-sized drones. Drones provide navigational interlocation services, which are made possible by the Internet of Things (IoT). Drone networks, on the other hand, are subject to privacy and security risks due to design flaws. To achieve the desired performance, it is necessary to create a protected network. The goal of the current study is to look at recent privacy and security concerns influencing the network of drones (NoD). The current research emphasizes the importance of a security-empowered drone network to prevent interception and intrusion. A hybrid ML technique of logistic regression and random forest is used for the purpose of classification of data instances for maximal efficacy. By incorporating sophisticated artificial-intelligence-inspired techniques into the framework of a NoD, the proposed technique mitigates cybersecurity vulnerabilities while making the NoD protected and secure. For validation purposes, the suggested technique is tested against a challenging dataset, registering enhanced performance results in terms of temporal efficacy (34.56 s), statistical measures (precision (97.68%), accuracy (98.58%), recall (98.59%), F-measure (99.01%), reliability (94.69%), and stability (0.73).

## 1. Introduction

The environmental ubiquity of smart technologies enables applications with elevated performance and efficiency [1]. It provides enhanced capabilities by object configuration in the smart city [2]. Drone technology has recently led to the development of miniature drones such as quadcopters and micro drones [3]. These small drones have the advantage of being able to easily enter any infrastructure for tracking numerous domains including industrial surveillance and disaster response, search and rescue, military usage, accurate agriculture, shipping, and delivery. Drones or *unmanned aerial vehicles (UAVs)* are air-bound aircraft that do not have human operators [4]. They can be used for aerial photography, weather forecasting, and other purposes. UAVs are typically utilized by aerodynamics forces to supply them with facilities of controlling a machine from a distance [5,6]. There are comparable commercial applications that have influenced the features of various businesses and have impacted everybody. UAVs are useful vehicles for surveillance and monitoring since they are often capable of gathering airborne data and relaying it to the base station with ease.

### 1.1. Research Domain

The widespread adoption of drone technology has posed issues, in addition to security, in terms of liability, privacy, and regulation. Reduced-sized UAVs offer several advantages in shipping, distribution, and privacy such that drone technology has become a part of daily lives [7]. The security and privacy of these drones, on the other hand, are a major concern. Another developing subject of research in recent years has been the quest of conceptualizing smart drones by the addition of IoT sensors that can be embedded in small drones. Drones may be made more helpful and effective by using a collection of technologies such as sensors, transmitters, and cameras for a variety of various and sophisticated applications. Small drones are giving the defense and civic industries new opportunities. However, due to a lack of adequate design, tiny drones are vulnerable to privacy and security risks. A network of drones (NoD) provides novel pathways while posing new hindrances in terms of security and privacy.

### 1.2. Research Motivation

**Figure 1:**
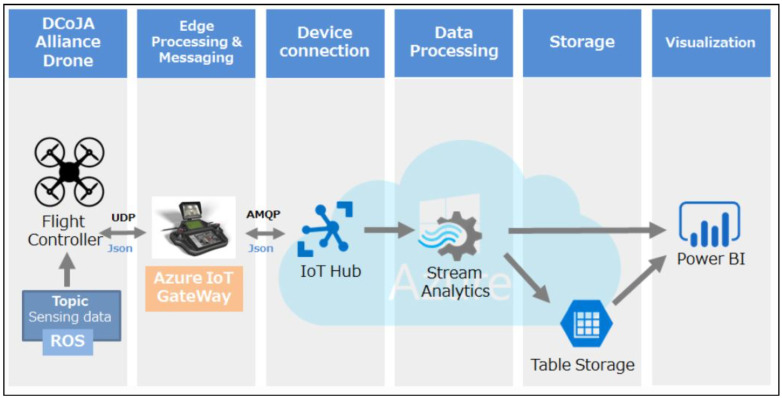
UAV architectural modules.

The basic architecture and design of NoD must be modified to provide enhanced security and reliability. Conventionally, a typical drone’s structure was built on a layered architecture, as depicted in Figure 1. The drone module is the initial module in a typical modular architecture of industrial drones, where a copter is embedded with a camera. Through an IoT gateway, the smart drone is connected to the terrestrial network [8]. IoT gateways, such as a cloud-equipped network of a terrestrial station, play an important role in providing communication in the scenario. The information acquired at the IoT hub is forwarded to the computational module, which examines the information stream. At the storage module, the results of the computational analysis are saved in the database and are further transmitted to the visualization module for in-depth analysis. Moreover, the platform is compatible with the Microsoft Azure cloud environment. On the contrary, one drawback is its lack of functionality to support data security and privacy. The *Internetwork of Drone Things (IDT)* is a contemporary concept that combines IoT and drones, allowing drones to connect to a network of IoT devices. The idea of the IDT is presented in the current study for addressing security and privacy issues.

#### Problem Definition

IDT security is one of the major concerns for the successful deployment of the NoD. With the innovations in the domain of ML, numerous opportunities can be explored for the secure deployment of the NoD. The current paper addresses the concern by exploiting ML techniques for the comprehensive deployment of the NoD network.

### 1.3. Major Contribution

The presented work presents the augmentation of IoT and drones to create a smart drone vision with data analysis capability. Moreover, blockchain technology is utilized to ensure security and privacy in smart drones. *Drone module*, *edge computational module, security module, transmission module, processing module, storage module, and visualization module* are the seven modules in the proposed framework. The following are the key objectives of the comprehensive IDT-based framework:

1. A comprehensive modular framework is proposed to ensure privacy and security in the drone network.
2. The proposed framework highlights vital details, analysis, hardware modules, and security techniques using machine learning (ML) models.
3. To manage security attacks, the proposed framework uses IoT sensors, drone data, and networking data.
4 The proposed model is evaluated on two challenging datasets in which enhanced temporal efficacy (34.56 s), statistical measures (precision (97.68%), accuracy (98.58%), recall (98.59%), F-measure (99.01%), reliability (94.69%), and stability (0.73) are registered.
5 The experimental findings show that the proposed model is generalizable and resilient.

#### Paper Organization

Section 2 highlights the state-of-the-art research in security, vulnerabilities, and attacks against drones and IoT systems. The proposed research framework for safe drone systems is described in Section 3. Moreover, secure authentication and access control techniques for drones are discussed. The experimental implementations and results are presented in Section 4. Section 5 discusses the limitations and future work directions. Finally, the paper is concluded in Section 6.

## 2. Literature Review

Drones are typically used for defense and military objectives. Drones range in size from 200-foot war machines for military use to micro flying nodes. Drone size is a significant element in terms of usage and functions. The range of flight varies from a few feet to a military drone that can cover a haltless 16,999 miles of land cover. The maximal areal duration varies depending on the surface area, scenery, and altitude. The flight’s altitude fluctuates from a few meters to 65,000 feet. The current study identifies the security-related 51 publications out of 189 research publications covered by the Web of Science (WoS) database from 2015 to 2021. The keywords used for the search query include “security”, “machine learning”, “drones”, and “Internet of Things”.

### 2.1. Drone Security Threats

Drone security is divided into numerous types and tiers based on its intended usage, size, and control methods. The Wi-Fi communication protocol (IEEE 802.11) is used by the drone in the majority of circumstances [9]. A Wi-Fi network and associated terrestrial hubs are part of the drone infrastructure, and both are exposed to cybersecurity attacks. Drones can be hijacked due to the lack of encryption mechanisms in machinery, as presented by Yin et al. [10]. Koslowski et al. [11] depicted that attacks including man-in-middle have a wide range of 3 km which can lead to hijackings. Ozmen et al. [12] showed that in the military business, IDT is becoming increasingly popular. One major issue is that it was designed to ensure privacy and security. Khan et al. [13] showed that data protection, loss, and cryptographic procedures are vital privacy concerns. Many researchers have found security risks in recent years. Sensor-specific, protocol-specific threats, and corrupted components are vital threats. Table 1 shows an overview of the literature on different categories of attacks. The prior literature has mostly concentrated on identifying cybersecurity concerns in drones. In many situations, the remedy to these risks is not considered. The utilization of cryptographic data during communication to the terrestrial station by cryptography mechanism for secure transmission was the major objective of Ranjitha et al. [14]. Li and Bai [15] stated that due to their compact size and reduced weight, mini-drones have been the center of attention among researchers in recent times. The small-sized drone poses a danger to the privacy of public and government information. Many additional studies of Cabassi at al. [7], Aldhyani et al. [3], and Tuli et al. [16], have looked at the security dangers and issues that drones pose. Khan et al. [17] presented an effective and intelligent edge-assisted IDT authentication scheme that secured the NoD. Maghazei et al. [18] introduced a drone data security surveillance framework for a manufacturing environment. A gas-emission industrial drone framework was proposed by Kapoutsis et al. [19]. Drones are mostly utilized for surveillance in the agriculture and security industries. In the last decade, the analysis of cyberthreats for drones has been a challenging research domain. Nguyen et al. [20] discussed smart city drone applications and the privacy risks that come with them. The cybersecurity threats posed by drone networks, along with limitations and future directions, were discussed by Kumar et al. [21]. Aydin et al. [22] suggested that blockchain could be used for secure data transfer via IoT-equipped drones. The system had to manually identify the types of threats and severity. Drones must be part of an effective, smart, and secure system for identifying cyberthreats and taking preventative actions to ensure data protection. Aloqaily et al. [2] identified security risks related to industrial and commercial drones, along with challenges and solutions. For secure drone data delivery, Maddikunta et al. [23] used key agreement and key-enabling information to solve the problem of device authentication. In Saha et al. [24], the use of IoT-based drones in agriculture was considered. As stated by Lyu et al. [25], drone hijacking, drone data theft, and UAV theft are all problems that commercial drones confront. In Jares et al. [26], countermeasures and solutions to security challenges were presented. Another issue concerning UAVs is GPS spoofing, which requires a safe, secure, and authentic solution. Talaei et al. [27] detailed several experiments on hacking and controlling UAVs. A detailed comparative analysis of drone attacks is depicted in Table 2.

### 2.2. Drone Security Using Machine Learning

Supervised, unsupervised, and semi-supervised learning are the three primary types of ML approaches. Many researchers have used ML models to cope with cyberthreats in communication networks [34], IoT networks [35], and cloud computing [36]. Vedula et al. [37] used RF and LSTM (autoencoder) to combine a learning methodology with a self-adaptive model to identify DDoS attacks using two features. Hosseinzadeh et al. [38] suggested a probabilistic strategy for detecting and controlling an actuation attack in a limited cyberphysical system. There has been limited research on cyberattacks in drone networks using ML algorithms. Conspicuously, in terms of drone security, an access control mechanism is proposed in the current research. Table 3 summarizes state-of-the-art works on the application of ML in wireless network security solutions. A huge number of papers have been published on privacy and safety problems of drone data security from 2010 to 2020, according to a comprehensive literature analysis. The majority of the research explores cybersecurity difficulties, applications, and issues. Moreover, data protection, drone hijacking, and spoofing are also considered. Many studies have identified the problem domain but have not offered a feasible remedy for resolving these issues. Bera et al. [39] proposed a blockchain-based solution for data security during communication via IoT-enabled drones. The presented technique involves manual detection of attacks. A device-based authentication system was presented; however, it was not suited for an IoT-based network of drones. By presenting a technique that addresses cyberthreat concerns to ensure drone adaptability in the industrial sector, there is an open research challenge in the field of building a secure IDT.

For drone security, a sophisticated and smart framework is necessary for data analysis from attacks and ensuring drone security by provisioning vital actions. Previously, artificial-intelligence-inspired techniques for mobile-based networks were proposed in the field of cyberthreats, but not for drone-based security. The current paper proposes a machine-learning-inspired technique for authentication, security, and control access of drones. Based on the comprehensive literature review, Table 4 presents a comparative analysis with the state-of-the-art literature review in the current domain.

## 3. Proposed Framework

**Figure 2:**
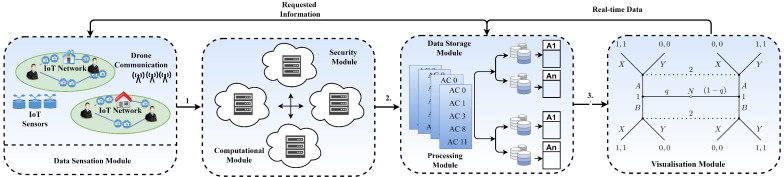
Proposed modular framework: conceptual view.

The proposed framework focuses on strengthening the cybersecurity of drones and IoT devices. The current study contributes to improving the fundamental framework of drones, particularly small-sized drones, to assure dependability and security in the face of typical cyber-risks, privacy vulnerability, and interception threats. The suggested framework is proposed as a modular approach, with each module dealing with different security challenges and techniques of analysis. In addition to the typical drone operations, the modular method includes data security and analytic tools. Furthermore, the modular architecture simplifies its implementation as well as future modification and development. The suggested technique increases drone data security by employing machine intelligence through the usage of ML models. Figure 2 depicts the conceptual diagram of the suggested framework. In the defense industry, miniature drones are provisioning new research possibilities. However, due to a lack of adequate design, small-sized drones are vulnerable to privacy and security risks. Modifications in the IDT and IoT provide novel possibilities while also posing new concerns in terms of data privacy and security. In respect of data privacy considerations, the current architecture is not yet safe and dependable.

### 3.1. Procedural Steps

Based on the aforementioned aspects, the procedural steps of the proposed approach are discussed ahead.

1 In the initial step, the drone module acquires the ubiquitous information using numerous embedded IoT sensors and camcorders.
2 The acquired data is forwarded to the edge computational module for local analysis.
3 Based on anomaly elimination using quantile regression [42], level-1 of physical data security is ensured.
4 Once security is ensured, the data are forwarded to the regional data hub for storage and application purposes.
5 Before deployment of the application, the acquired data are processed for level-2 security using the hybrid ML technique of logistic regression and random forest. The acquired data segments are generated by the sensors equipped over drones in heterogeneous format, which is classified as *normal or attacked* data segment using ML technique.
6 Moreover, the data are stored in the NoSQL data format repository for effective application-specific computation.
7 Finally, the visualization of the drone attack is performed over the Microsoft Azure framework.

Conspicuously, the aforementioned procedural steps depict the two-level security procedure for the presented architecture. A detailed description of each module is presented ahead.

### 3.2. Modules

The modular framework of intelligent drones is modified by augmenting the security and privacy modules. Additionally, the computational module is updated with ML components.

#### 3.2.1. Drone Module (DM)

**Figure 3:**
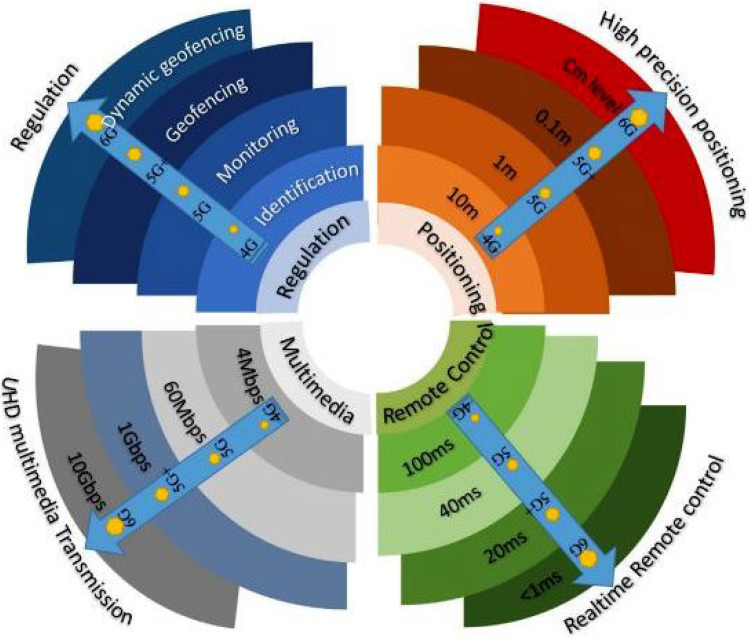
6G technology for UAV communication.

The first module in the presented modular framework of the industrial drone is the *drone module (DM)*, which includes a camera coupled to a quadcopter or other micro drone. IoT sensor data is used to update this layer. An altitude sensor, GPS sensor, radar, and camcorder are smart sensors employed. It is the first step in the architecture that has been proposed. The current module is capable of recording, sensing, and transmitting data to the second module. An unmanned aircraft vehicle (UAV) is involved in the current module for flight operation and data sensation. The UAV is made up of two components: (1) *terrestrial controller*; (2) *communication link*. A DJI Phantom 3 drone with a modified controller and transmission network is employed in the suggested design. IoT devices are embedded in the drone in the proposed architecture. For the communication link, state-of-the-art research presents novel benefits and specifications of using 6G technology, as shown in Figure 3 [45].

#### 3.2.2. Edge Computational Module

**Figure 4:**
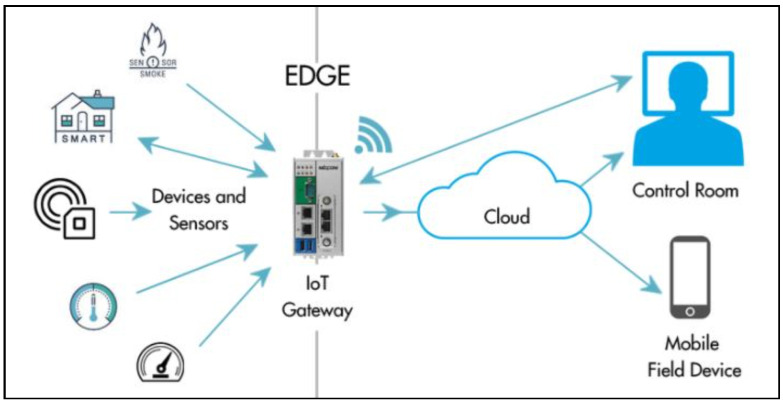
Edge computational module: generic view.

UAV drone data are transmitted to the security module where data authentication is confirmed. The current module, also known as the cloud module, is responsible for data transfer and transmission to the next station. There are several wireless communication gateway device techniques available. Wi-Fi communication is a type of wireless communication that sends data at a high rate. The device-to-cloud communication is efficiently handled by the edge processing layer. Data protection, cashing, and flooding are all handled by this layer. For cloud communication, the proposed study employs the Azure IoT gateway. Figure 4 depicts the architecture of the IoT gateway Source: https://www.digi.com/solutions/by-technology/edge-computing, accessed on 24 February 2022.

#### 3.2.3. Security Module

The current module plays a significant role in delivering device authentication and safe access control in the form of level-1 security. Data security and privacy, which are critical components of the presented IoT architecture, are implemented in the current module. However, there are a variety of privacy issues that might arise, including

1 Danger to physical privacy.
2 Threat to behavior privacy.
3 Threat to location privacy.

Capturing someone’s property is connected to physical privacy. If a third party is suspiciously monitoring drone data, personal information about someone’s property might be compromised. An unauthorized individual capturing a location is referred to as a location privacy threat. The surveillance of someone’s actions and behavior by an unauthorized individual is referred to as a behavior privacy threat. Authentication systems and protocols must be used to address such security threats. Unauthorized individuals employ a variety of security breaches to create security hazards. The most prevalent threats are an intrusion, spoofing, jamming, and denial-of-service (DoS) attacks. Device anomaly detection is maintained in the proposed architecture utilizing quantile regression [33] to determine and inform consumers of attack attempts.

#### 3.2.4. Transmission Module

**Figure 5:**
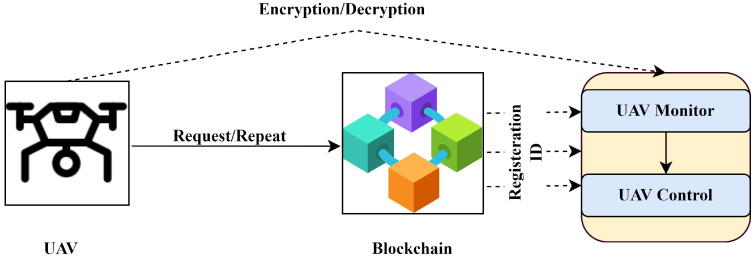
IoT security module: generic view.

IoT gateways are critical for establishing a communication link to a base station’s cloud-based IoT Hub. An automated module for security orchestration is emplaced to ensure authentic device connection. The IoT Hub facilitates communication between IoT applications and IoT devices. In an IoT network, the IoT hub allows messages to be passed between IoT devices and cloud systems. This is a two-way conversation. Security precautions for only authorized devices are created in this tier. The registration and encryption procedure for networked devices is depicted in Figure 5. Sensor data and network data are sent to the blockchain client, which ensures data integrity and saves the data on a cloud database. A fundamental security technique ensures real-time device and IoT safety.

#### 3.2.5. Processing Module

The information from the IoT gateway is subsequently transmitted to the *processing module*, which analyzes the drone’s acquired information. An ML-inspired technique that performs effective computation and aids in feasible cloud-based storage is deployed in the current module. Several ML algorithms are appropriate for different conditions and data needs. The goal of the current study is to employ an intelligent ML method for device authentication. An intelligent ML technique of hybrid logistic regression and random forest (LRRF) makes up the current module as the form of level-2 security. The IoT gateway authenticates devices by utilizing the drones’ timestamp data for a set period. The model is trained and tested using data from drone flights. The model is first trained and then tested to see if it is sufficiently sophisticated to recognize the malicious activity. If the data are inconsistent, the proposed framework will be notified and prevented from cloud connectivity. If a drone’s conduct is vulnerable, it is discovered right away, and unlawful access is prohibited thanks to machine intelligence. Flight operations are linked to several security risks. The most prevalent type of danger is a man-in-the-middle attack, which happens when a third party hijacks and controls the drone. When an unauthenticated individual acquires drone control, fraudulent information might be broadcast.

#### 3.2.6. Probabilistic Ranking Strategy

For drone security, a sophisticated and intelligent system is required that can study attack data and maintain drone security through proactive actions. To develop a trustworthy system, a safe NoD depends on security, reliability, and accuracy. Earlier ML models for WSN and mobile networks were only presented in the field of cybersecurity. The current paper proposes an ML-based technique for drone security authentication and control access approaches. Cybersecurity frameworks are analyzed utilizing numerous measures. The objective of employing such metrics is to handle numerous performances in a system’s cybersecurity. In the current study, the following cybersecurity measures are analyzed to assess the proposed system’s performance.

1. Jamming.
2. Denial of service (DoS) attacks.
3. Exposure to cybersecurity threats from drones.
4. Malicious attacks.
5. Spoofing.

**Figure 6:**
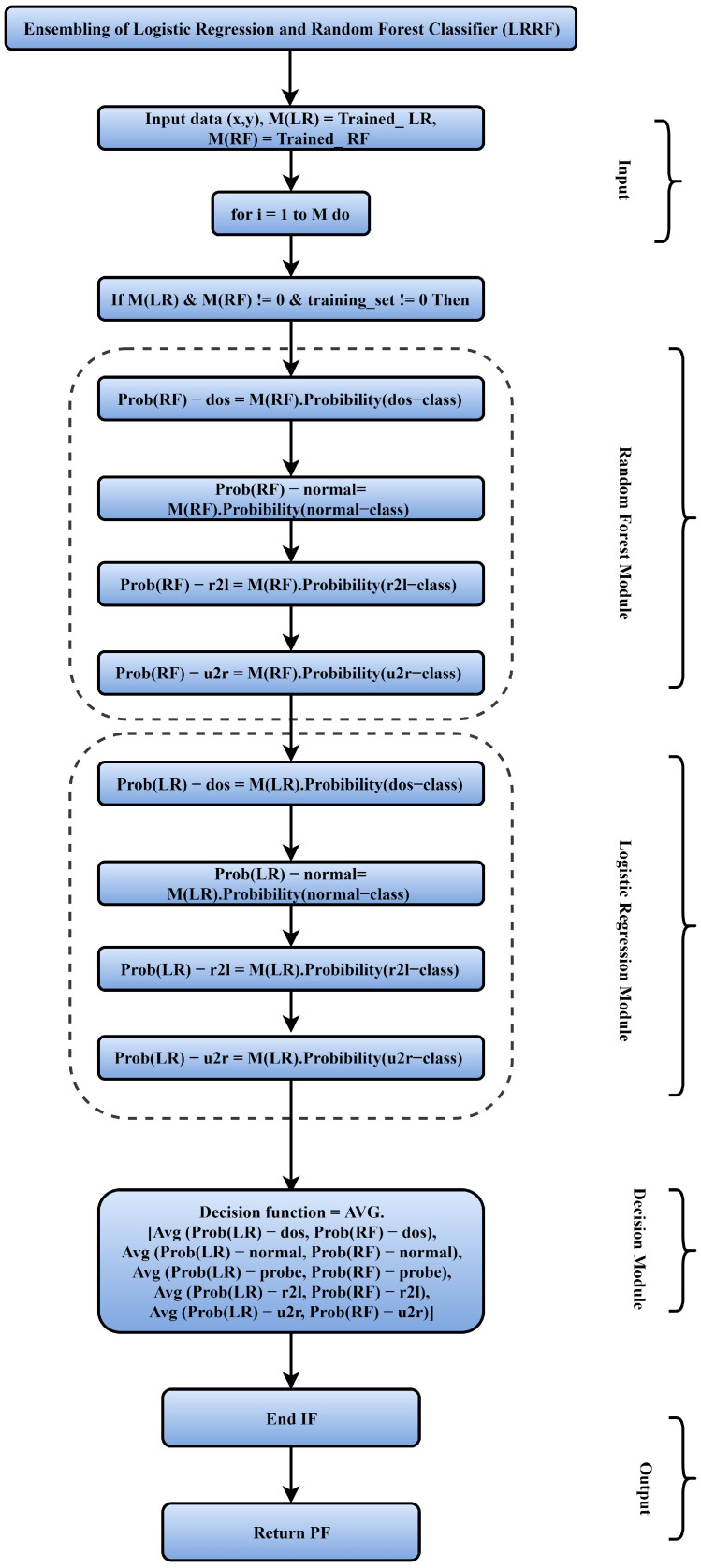
Proposed technique flow chart.

A key contribution of the current research is an ML-based technique for secure security and access control for IDT. The current research intends to address vital research challenges by securing drones against key cyberthreats. It is a helpful monitoring tool for both commercial and industrial applications. The suggested design of the drone security system consists of seven modules, as stated earlier. Before communication to the transmission module, drone module data are transferred through the security module. Data are protected from security attacks at the security module by employing ML models, which send a mobile warning when a vulnerability is detected. Moreover, two types of datasets are classified by the ML techniques, including *normal* data instance and *attacked* data instance. The proposed ML model performs effective identification of vulnerable data segments for ensuring data protection at the storage level. Specifically, the current research uses a *probabilistic ranking classifier* that combines logistic regression and random forest, and it outperforms other intrusion detection approaches individually. The suggested probabilistic ranking classifier’s operation is described in Figure 6. The detailed stepwise procedure is depicted ahead.

##### Stepwise Procedure

In the proposed technique, the input data (x,y) are fed to the ML model. The proposed hybrid model is trained using logistic regression and random forest (M(LR) and M(RF)). For all data attributes in a given time window δt, four categories of attacks have been identified, namely, denial of service (DoS), remote to user (r2l), user to root (u2r), and probe attack (Table 4). The RF module assesses the data for the categorical attack, which are recursively analyzed using the LR module of the model. The decision function computes the average probability of vulnerable data. Mathematically, the decision function (PF) is computed as PF = ArgMax($\sum_{j=1}^{m}LRj,\sum_{j=1}^{m}RF_{j}$) where $\sum_{j=1}^{m}LR_{j}$ and $\sum_{j=1}^{m}RF_{j}$ are prediction-based results. *Soft ranking* and *hard ranking* are the two types of classification techniques. The outcomes estimated by classifiers are taken into account in the hard ranking type. The soft ranking category, on the other hand, calculates each classifier’s percentage weight. To obtain the conclusion, the model predicts the class probability for each record, multiplies it by the classifier weight, and averages it. The presented model is trained to utilize the hybrid logistic regression and random forest classification (LRRF) technique. The trained model is utilized to certify engendered airplane routes. To assess performance parameters in terms of accuracy, precision, and recall, the challenging dataset was considered. *Precision* is the % of true correct predictions, while *recall* is the % of accurate predictions that are incorrect in reality.

#### 3.2.7. Storage Module

**Figure 7:**
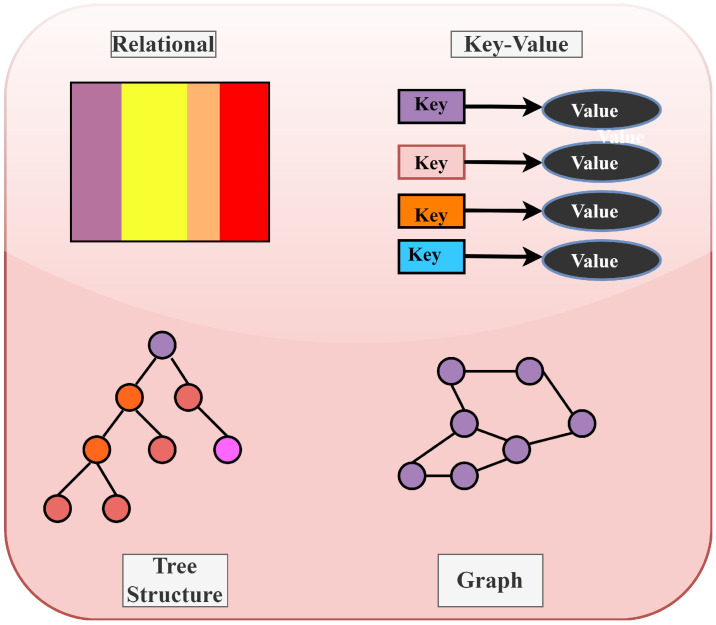
No SQL data structures: generic view.

In the *storage module*, the computational results created by the processing module are stored in a database. The findings generated by drones at the drone layer are stored in a cloud-based NoSQL database. IoT sensor data, as well as network and drone information, are included in the data. The NoSQL database stores data without using a schema, making it simple to access and retrieve data rapidly. With this method, a vast amount of data may be saved. NoSQL databases are self-referential, making them more useful than SQL databases. The common storage architectures utilized in such databases are depicted in Figure 7 Source: https://www.guru99.com/nosql-tutorial.html, accessed on 21 February 2022.

#### 3.2.8. Visualization Module

**Figure 8:**
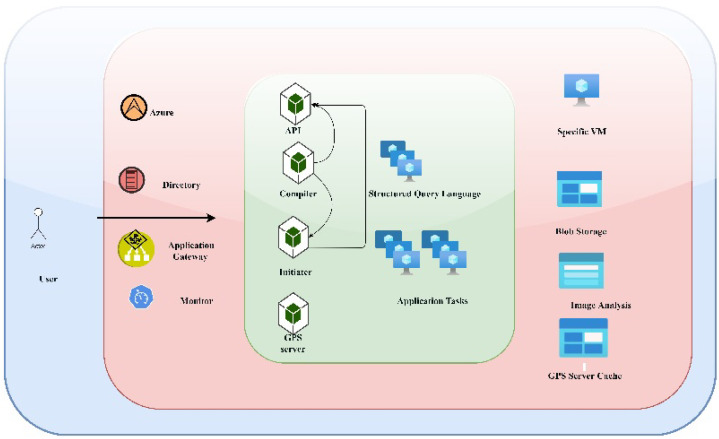
Microsoft Azure computing: generic view.

Data may be monitored using a variety of tools and services using the *visualization module*. The current framework makes use of Microsoft Azure for storing and gateway services. A mobile application is used to view the visualization layer’s results, which display the intelligent model’s predictions about a drone’s security level. The intelligent LRRF Bayes model is used to detect drone attacks. The framework of business intelligence employing data analysis, stored in the database, is shown in Figure 8 Source: https://docs.microsoft.com/en-us/azure/architecture/reference-architectures/containers/aks-microservices/aks-microservices, accessed on 24 February 2022. *Microsoft Azure Kubernetes services*, a tool for business intelligence modeling and results presentation, is utilized for visualization.

### 3.3. Hardware Components

The hardware resources utilized in the experimental simulation are both readily available and inexpensive. An Arduino 2670 enabled with Wi-Fi was utilized as a computational node to store data from IoT devices.

#### 3.3.1. DJI Phantom 4 Pro Drone

**Figure 9:**
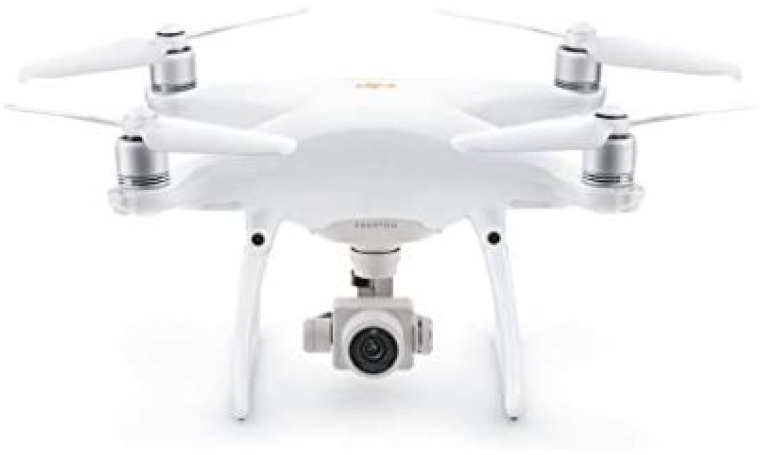
DJI Phantom 4 Pro drone.

UAVs currently come in a variety of designs and sizes. These shapes also govern how a drone works in terms of working style and size. The suggested framework makes use of a compressive Phantom 4 Pro aerial vehicle, which is manufactured by DJI. It is operated using a proprietary controller that can be accessed remotely from a distance. Figure 9 shows the incorporated drone model Source: https://dronedj.com/2022/01/05/dji-phantom-4-rtk-drone-firmware-update/, accessed on 24 February 2022.

#### 3.3.2. Sensor GPS

**Figure 10:**
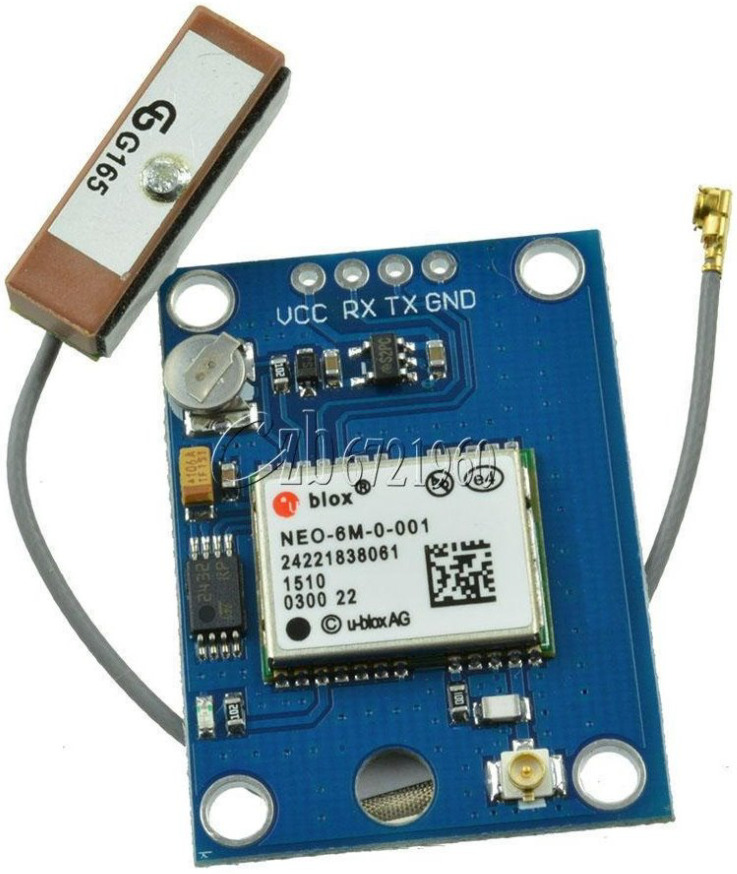
GY-GPS6MV2 module.

The GY-GPS6MV2 is a GPS receiver made up of an NEO-7N chip and an electric circuit. It is made up of a battery along with an LED display. When it transmits GPS data across satellites, the light turns on. A sensitivity of roughly 161 dBm is also provided by this sensor module. The incorporated GPS sensor is shown in Figure 10 Source: https://www.amazon.in/Generic-GYGPSV3-M8N-GPS-Module-GY-GPS6MV2/dp/B06ZZBZ3QP, accessed on 24 February 2022.

#### 3.3.3. Radar Sensor

This is utilized to monitor and identify objects from distance. Electromagnetic radiation is sent by these sensors toward objects and target locations. When compared to optical sensors, these sensors can identify things with greater precision. Radar sensors can be replaced by accelerometers. An HC-SR04 ultrasonic proximity sensor is employed in the suggested system for this purpose. A radar sensor calculates the patterns of objects.

#### 3.3.4. Wireless ZigBee Transmission

**Figure 11:**
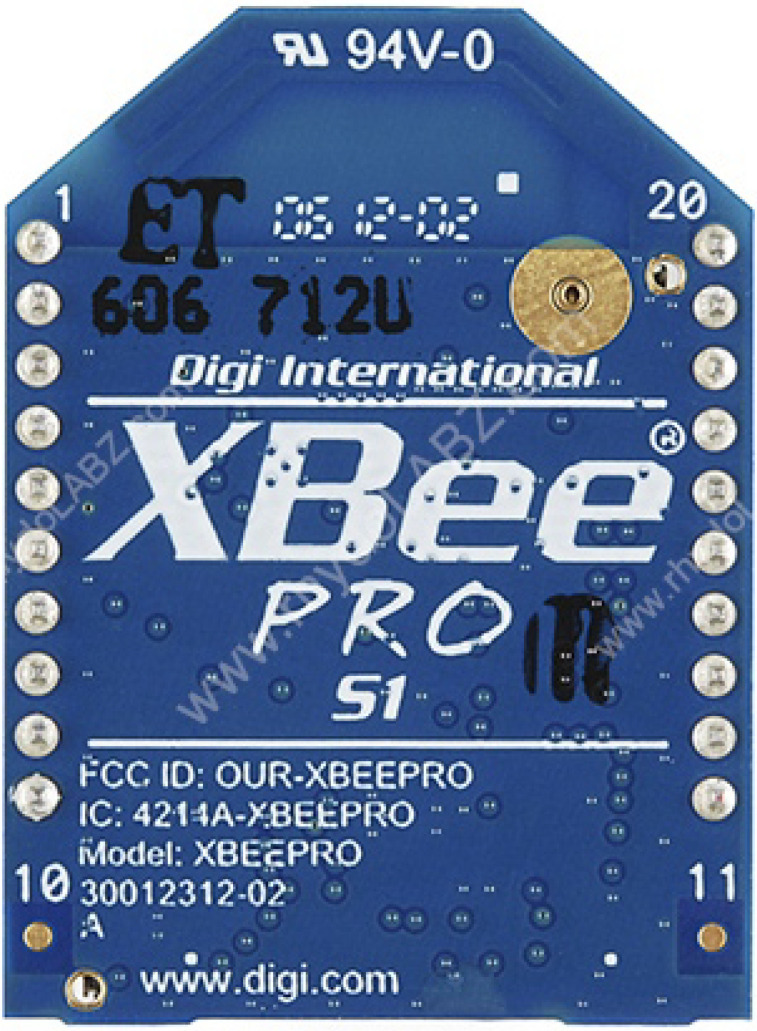
ZigBee module.

Because of the attributes, analogies, and digital information transmission capability, ZigBee wireless technology is presently employed. The XBee Pro S1, which can transmit data over a long range, is employed in the current study. The hardware of this module is shown in Figure 11.

#### 3.3.5. BMP180 Pressure Sensor

**Figure 12:**
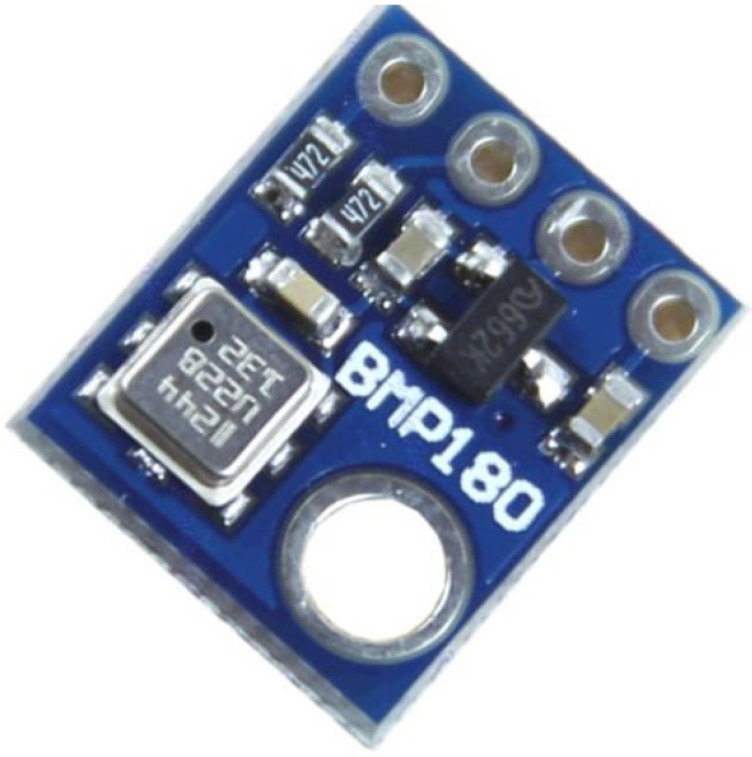
BMP180 pressure sensor.

This gives altitude and pressure measurements for a specific place while using minimal battery power. It is modest in size and efficient in terms of precision. The pressure sensor module has been calibrated by the manufacturing unit, making it more precise than alternative sensors. The hardware of the pressure sensor is shown in Figure 12.

## 4. Experimental Assessment

The compressive description of the proposed system was discussed in the preceding section. The current section encompasses the presented framework’s implementation analysis.

### 4.1. Dataset

The experimental simulation was carried out using time-sensitive data. KDD intrusion detection attributes drone data and GPS characteristics of altitude, latitude, and longitude were included for simulations. The proposed model for intrusion detection and cybersecurity attack prediction was assessed utilizing the challenging datasets. The detailed classifications are listed in Table 5. Numerically, more than 2521 data instances are considered in varied phases for detecting the efficacy of the presented technique.

### 4.2. Incorporated Machine Learning Technique

ML has made a significant contribution to the improvement of probabilistic ranking-based predictions. For rating classification, there are a plethora of ML classifiers. The Python Scikit-learn module contains a wide number of ML classifiers. It is an open-source library with a wide community of users. All classifiers in the current work, including gradient boosting model, stochastic gradient classifier, random forest, naive Bayes, extra tree classifier, and logistic regression, were implemented using the Scikit-Learn module. Each of these is discussed in detail ahead.

1. *Random forest (RF)* is an ensemble learning technique that uses decision trees (DT), which are also known as estimators, to classify data. The probabilistic ranking is used to combine the results of various trees to produce better results. The bagging technique is used to train trees using bootstrap samples. To evaluate the model’s performance on test data, all trees are created in the same way. The decision tree with the lowest error rate is given a larger weight, resulting in a lower likelihood of making an inaccurate estimation.
2. *Decision tree (DT)* is a multi-variable ML technique for text categorization that is widely used. It is used to forecast certain data values. It divides data into branching segments, which are utilized to construct an inverted tree with internal nodes, the root node, and leave nodes. The method is nonparametric, which means it can effectively assess complex data without imposing a sophisticated framework.
3. *Logistic regression* is a mathematical strategy to predict outcomes; it examines data and works on numerous factors. It is a low-variance, basic but efficient approach that is commonly used in classification. This model may also be used to extract features. Stochastic gradient descent makes it simple to update with fresh data.
4. The *naive Bayesian classifier*, which makes independent assumptions among predictors, is based on Bayes’ theorem. It is simple to put together, with simple iterative parameter estimation. As a result, it is thought to be ideal for huge datasets. Despite its simplicity, it produces excellent results and outperforms other, more advanced classifiers.
5. In text categorization, the *support vector machine (SVM)* is widely used. By maximizing the marginal distance, it draws hyperplanes that split classes. In binary classification, the SVM hyperplane separates the text into two separate groups. It is simpler and less complicated than deep learning approaches, and it is easy to understand. SVM is also commonly used to detect intrusions.
6. The *multilayer perceptron (MLP)* is a basic deep learning model that can classify data rather well. It is a layered model, with neurons representing features in the initial layer and hidden layers processing incoming data and feeding them to the output layer represented by neurons. The numbers of hidden layers and neurons are chosen optimally to obtain the best outcomes. The model is trained using the hyperparameter values to increase classification training efficiency. Backpropagation, which is based on gradient descent, is commonly employed to assess MLP weights. The *rectified linear unit (ReLU)* is employed in hidden layers, while the *sigmoid* is utilized as an activation function. Sigmoid (G(x))= $\frac{1}{\left(1+e^{-x}\right)}$.

The findings of several classifiers are pooled in ranking classifiers, and the ultimate choice is determined based on ranking.

### 4.3. Experimental Results

The list of suggested sensors and algorithms, as well as the testing, are described in depth in the previous section. The outcomes of the model, as well as the experiment, are discussed in this section. The findings for the mobile system, which includes the drones’ security status and the IoT network detected by machine learning, are shown. The performance of the models is compared using four assessment metrics in the current study in terms of temporal efficacy, statistical performance, reliability, and stability. Specifically, for statistical parameters, accuracy, precision, recall, and F-measure are estimated. These measurements may be calculated with the aid of the confusion matrix. False positive (*FP*), true negative *(TN)*, true positive (*TP*), and false negative (*FN*) are the constituents of the confusion matrix. The mathematical formulations are depicted ahead.

Accuracy = $\frac{TP+TN}{TP+TN+FP+FN}$

Precision = $\frac{TP}{TP+FP}$

Recall = $\frac{TP}{TP+FN}$

F1-Measure= $\frac{2*Precision*Recall}{Precision+Recall}$ = $\frac{2*TP}{2*TP+FP+FN}$

### 4.4. Temporal Efficacy

**Figure 13:**
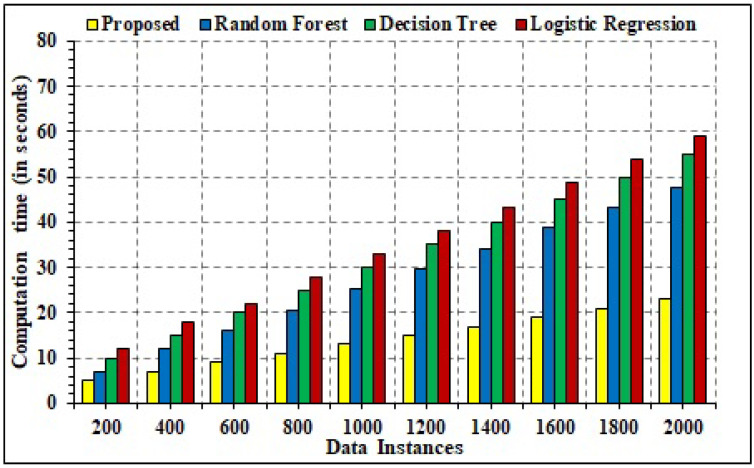
Temporal Delay.

The temporal efficacy deals with the delay computation for the varied number of data instances. For comparative analysis, three explicit ML models of random forest, decision tree, and logistic regressions are considered. However, it is important to mention that only the ML model is updated in the experimental simulation, while the remaining framework is unaltered. Temporal delay computes the running time of the ML model for detection of the vulnerable data instances for attack detection in the IoT–UAV scenario. The detailed analysis is depicted in Figure 13. It can be seen that in the varied number of data instances, the proposed model of hybrid LRRF is temporally more efficient as compared to other models. Specifically, the proposed model registers an average temporal delay of 34.56 s in comparison to 45.60 s for random forest, 67.34 s for decision tree, and 90.23 s for the logistic regression. It shows that the presented techniques are temporally more effective for the identification of attacks in comparison to other classification techniques. Moreover, computation time incorporates the total time from data generation to computation. However, for the comparative analysis, the trained model of LR and MF is considered for the comparative analysis. Since the trained model can classify vulnerable data in real time, the computation time is lower as compared to the conventional static model, which has to classify data statistically and therefore incorporates more time. Moreover, the computational time complexity for random forest is computed as O(n(tree)*m(try)*nlog(n)). Similarly, for decision tree and logistic regression, the computational complexity is computed as O(v(variables)*n (records) log(n)) and O(n(data points)d(size)). However, in the current scenario, the proposed model’s asymptotic computational complexity is computed as O(n(iterations)logn), which is theoretically better than comparative techniques. It can be concluded from the fact that the average of multiple classification techniques is utilized. Therefore, the temporal efficacy tends to be less in the generation of optimal results.

### 4.5. Statistical Analysis

**Figure 14:**
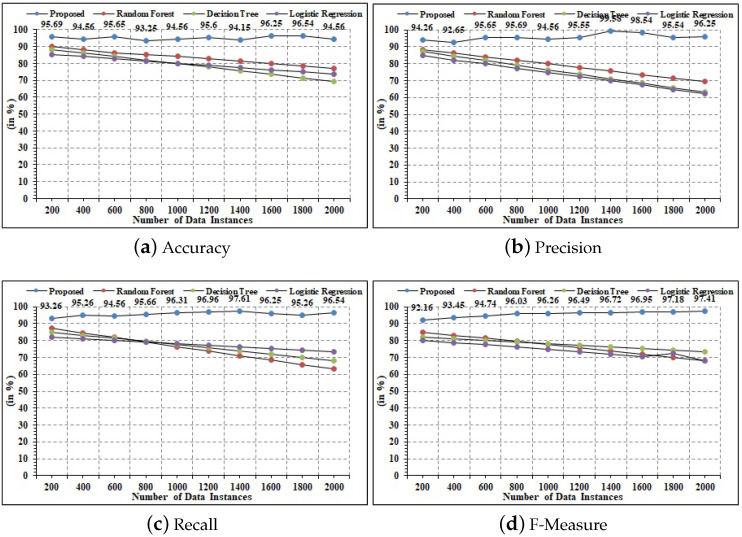
Statistical results.

The statistical findings in terms of performance parameters of accuracy, precision, recall, and F1-measure are discussed in the current section. The presented framework’s results are compared with state-of-the-art ML models that were used on the drone dataset. Explicitly, decision tree, random forest, multilayer perceptron, logistic regression, Naive Bayes, support vector machine, and random forest are the incorporated classifiers. All of the tests were carried out in Python using *Keras*, *SciKit-learn*, and *Tensorflow*. Data were divided into three categories of *jamming, spoofing, and denial-of-service attacks*. Table 6 demonstrates the detailed computation results of statistical parameters. The proposed technique acquired 98.58% accuracy, which is the highest accuracy score for threat detection in comparison to other techniques. This shows that the presented technique is optimal in the detection of attacks and classifying data instances in real time. Moreover, for precision analysis, the presented model was able to acquire the maximal value of 97.68% in comparison to state-of-the-art ML techniques. Moreover, logistic regression was the next ML which obtained the second-best precision measure of 96.25%. This can be deduced from the fact that the presented model is made of hybrid logistic regression, which has influenced precision computation. Similarly, for recall computation, it can be seen that the presented model attained an elevated value of 98.59%, which is comparatively better than ML other models in the current scenario. It depicts that the presented technique has the ability to estimated correct data instances which are vulnerable to attacks in the IoT–UAV scenario. Likewise, for the estimation of the F-measure, the presented model was able to register a maximal value of 99.01%, which shows the enhanced performance. Comprehensively, it can be stated that in the current scenario, the presented technique is statistically better. Figure 14a–d depict the detailed results for statistical measures for a variable number of instances. Moreover, it can be seen that the performance of the comparative ML technique is decreasing with an increasing number of instances. This is because the conventional ML technique uses the statistical approach for performance estimation. However, the presented techniques use iterative techniques to minimize the error in the computation. The iterative behavior ensures optimization of the computation. Moreover, with the increased number of data instances, the training accuracy of the proposed model increases, which increases the accuracy in the case of large data instances.

In addition to the aforementioned results, the presented model was compared for performance assessment for different datasets. The Performance analysis can be found in Table 7.

Specifically, two challenging datasets, NS-KDD [46] and KDD Cup 99 [47], were considered for estimating the accuracy of the proposed techniques. For comparative analysis, numerous hybrid ML models of principal component analysis (PCA) and multiple correspondence analysis (MCA), deep neural network model, and decision tree and random forest (DT–RF) are considered. The detailed results are depicted in Table 6. It can be seen that in all the three scenarios, the proposed model was able to attain maximal accuracy of 98.58%, 98.69%, and 99.01%. The tabular measures depict the performance over different datasets. Moreover, p-test was used to look for any discernible difference between the ML techniques. Specifically, *null hypothesis* was created to assume that there is no significant difference between the baseline and proposed techniques. The outcomes vary due to the nature of stochastic technique or assessment approach, as well as numerical precision variations. In the current scenario, the p-value is nearly 0.02, which is less than 0.05. As a consequence, it rejects the null hypothesis, meaning that there is discernible difference between the ML techniques. Henceforth, it can be concluded that in the current scenario, the proposed model is better and more effective in the detection of attacks.

### 4.6. Reliability

**Figure 15:**
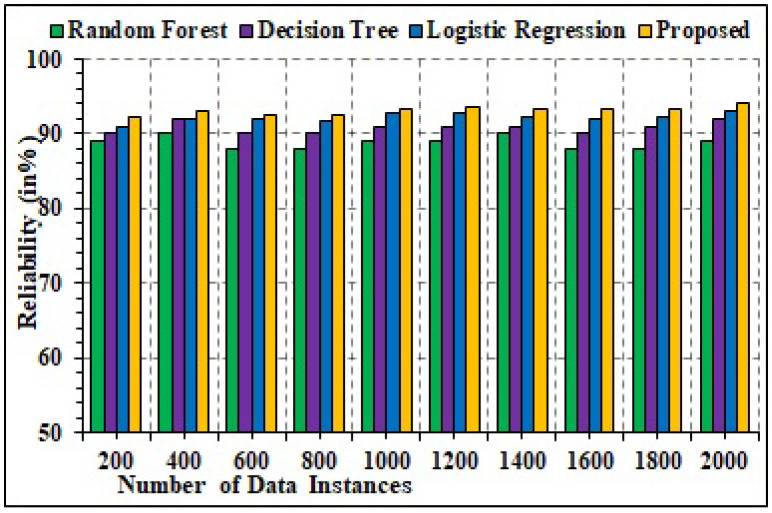
Reliability analysis.

Reliability is another parameter for performance assessment computed in the current study for detailed analysis of dependability. For comparative purposes, explicit random forest, decision trees, and logistic regression techniques are used. Figure 15 depicts the graphical results for the dependability analysis. It can be seen from data measures that the presented technique can acquire maximal reliability of 94.69%. In comparison to this, the random forest ML technique registered an average dependability value of 89.65%, decision tree acquired 90.02%, and logistic regression attained 91.26%. This can be concluded from the fact that in the current scenario, the proposed technique utilized the hybrid technique for estimating the attacks. Henceforth, elevated reliability measure is registered.

### 4.7. Stability

**Figure 16:**
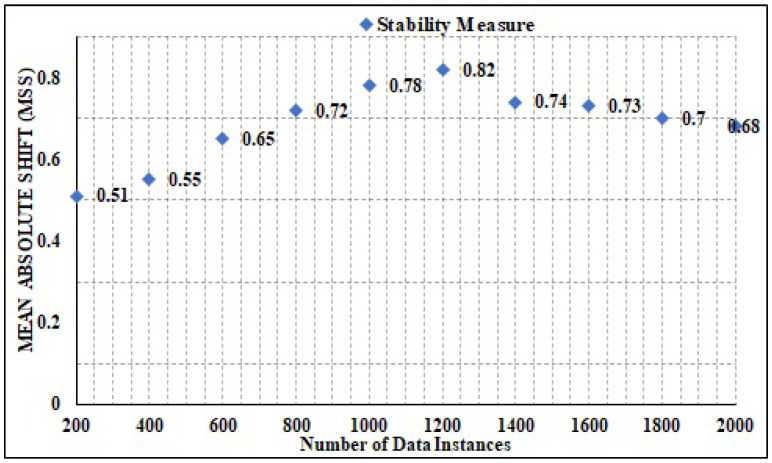
Stability analysis.

Stability analysis is another vital performance measure for estimating the effective result computation over the varied number of data instances. It is computed in terms of the mean absolute shift value. The range of the MAS value varies from 0 to 1. Specifically, 0 depicts a low stability measure that is not suitable for the model, and 1 depicts a maximal stability measure, which shows enhanced suitability of the techniques. The detailed results are computed and shown in Figure 16. It can be seen that the presented technique registered a minimal stability measure of 0.51 and maximal measure of 0.82, averaging 0.73. Henceforth, the presented technique has attained stability measure at an elevated level. It can be concluded that in the current scenario, the presented model is better and more stable.

### 4.8. Scalability Analysis

**Figure 17:**
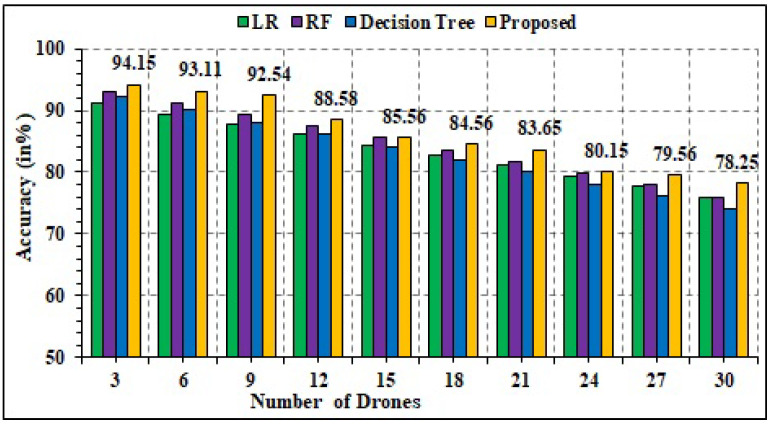
Accuracy analysis.

The scalability analysis is performed for the proposed model in terms of the number of drones. In other words, when the number of drones is higher, the system accuracy is computed. It is analogous to stability analysis; however, the difference lies in the fact that data identification is to be performed beforehand. Specifically, given N number of drones, the data generated by each drone are registered as *d_i_*, where *i* ∈ 1 to *N*. The bootstrapping technique is used for result computation for a large number of drones. Figure 17 shows the results of accuracy for different ML techniques. In the current scenario, the proposed technique is better and more effective. Specifically, the proposed model can register enhanced accuracy measures ranging from 94.15% for three drones to 78.25% for 30 drones. Henceforth, the proposed technique is more scalable compared to other techniques.

### 4.9. Cost Analysis

The cost analysis is performed for the proposed module for understanding the overall cost for reproduction. As mentioned before, the current framework incorporates numerous hardware components for deployment purposes. Conspicuously, the cost computation is performed based on the hardware as well as software entities utilized in the current study. Initially, the DJI Photon drone system embedded with the camcorder is used for acquiring ubiquitous data. The cost computation for data sensation is approximately USD 1985, having a 12 MP inbuilt camera, GPS, and pressure sensor. The communication module of ZigBee is used for data transmission with costing of USD 15. For data computation, the Raspberry Pi module is used, having the cost of USD 75 with 8 GB RAM. Finally, the Microsoft Blob storage for ML-based data analysis is used, having the cost of USD 0.15 per GB per month.

## 5. Limitations and Future Works

Every research has specific limitations that can be worked upon for future directions. The current work incorporates several limiting aspects that can be explored for future research. Some of the limiting aspects and future directions are depicted ahead (The Comparative analysis can be found in Table 8).

1. The proposed model is suitable for four categories of attacks only. This aspect can be explored for other types of UAV attacks, including DDoS and man-in-middle.
2. The application domain of the proposed model can be explored for deployment in different sectors for performance assessment.
3. The computation cost of the proposed model increases with a large number of data segments. It can be further improved using effective techniques.
4. Finally, the security-based network topology of drones is another aspect for future exploration.

## 6. Conclusions

Using a hybrid ML technique of logistic regression and random forest (LRRF), the current article provides IoT-assisted cybersecurity for drone-based networks. The presented framework uses IoT data from drones, sensors, and network information to create security-level patterns and identify security threats that exploit attack patterns. The presented technique can detect attacks on in-network data using the hybrid LRRF technique. The suggested system has been tested using a challenging dataset and has shown to be effective in detecting cyberattacks in real time. The model’s performance is measured in terms of statistical parameters of temporal efficacy (34.56 s), statistical measures (precision (97.68%), accuracy (98.58%), recall (98.59%), F-measure (99.01%), reliability (94.69%), and stability (0.73). The presented model exhibits its generalizability and robustness by appropriately detecting attack types. For future research, the suggested framework will be tried on different domains for intrusion detection.

## Figures and Tables

**Table 1:** UAV security comparative analysis.

Attack	Attributes	Reference
Protocol-based attacks	Security of communication link	[28]
Protocol-based attacks	Data confidentiality	[29]
Protocol-based attacks	Replay attack	[30]
Protocol-based attacks	Privacy leakage	[1]
Sensors-based attacks	GPS spoofing/jamming attack	[31]
Sensors-based attacks	Motion sensors spoofing	[27]
Sensors-based attacks	UAV spoofing/jamming attack	[31]
Compromised component	IoT security threats	[1]
Compromised component	Control/data interception	[32]
Jammers	Denial of service	[33]
Jammers	Stop packet delivery	[6]

**Table 2:** Drone cyberattacks (1: available; 0: not available).

Attack	Privacy	Conf	Int	Ave	Auth	Security Measures
Malware	1	1	1	1	1	Hybrid lightweight IDS
Social engineering	1	1	0	0	1	Raising awareness, training operators
Backdoor access	1	1	1	1	1	Hybrid lightweight IDS
Baiting	1	1	1	0	1	Raising awareness
Fabrication	1	0	1	0	1	Assigning privilege
Eavesdropping	1	1	0	0	0	N/A
Man-in-the-middle	1	1	1	0	0	Lightweight hybrid IDS
Wi-Fi aircrack	0	0	0	0	1	Lightweight IDS
Wi-Fi jamming	0	0	0	0	1	Frequency hopping, frequency range variation
Replay	0	0	0	0	1	Frequency hopping, time stamps
Ping-of-death	0	0	0	0	1	Frequency range variation
GPS spoofing	0	0	0	0	1	Return-to-base

**Table 3:** Data security for intelligent drones.

Attacks	Security Technique	Machine Learning Solution	Reference
Jamming	Secure offloading	Q-learning, DQN	[40]
Denial of service	Secure offloading	Neural network, multivariate correlation analysis	[41]
Intrusion	Access control	Naive Bayes	[42]
Malware	Access control	Random forest	[43]
Spoofing	Authentication	SVM	[44]
Traffic blockage	Authentication	Q-learning	[40]

**Table 4:** Comparative assessment (YY: available, NA: not available).

Comparative Works	UAV	ML Technique	M2M Communication	Cognitive Decision	Security	Real-Time	Performance Analysis	Numerical Quantification	Packet Evaluation	Statistical Analysis	Temporal Delay
[40]	YY	YY	YY	NA	NA	YY	YY	YY	NA	NA	NA
[43]	YY	YY	YY	YY	YY	NA	YY	NA	NA	NA	YY
[44]	YY	YY	NA	NA	YY	NA	YY	NA	YY	NA	NA
[41]	YY	YY	NA	NA	YY	YY	YY	YY	NA	NA	NA
[42]	YY	YY	YY	NA	YY	YY	YY	NA	YY	NA	NA
[27]	YY	YY	NA	YY	NA	NA	NA	YY	NA	NA	NA
[23]	YY	NA	YY	YY	NA	NA	YY	NA	NA	YY	NA
[24]	YY	NA	YY	NA	NA	YY	YY	NA	YY	NA	NA
[2]	YY	YY	YY	NA	YY	NA	NA	NA	NA	NA	NA
**Proposed**	YY	YY	YY	YY	YY	YY	YY	YY	YY	YY	YY

**Table 5:** Attack type and categories.

Attack Class	Type
DOS	land, back, pod, smurf
R2L	ftp_write, imap, multihop, phf, spy
U2R	buffer-overflow, perl
Probe	ipsweep, portsweep

**Table 6:** Dataset classes.

Category	Detail
Normal	Connections are generated by simulating user behavior.
DoS attacks	Use of resources or services are denied to authorized users.
Probe attack	Information about the system is exposed to unauthorized entities.
User to remote attacks	Access to account types of administrator is gained by unauthorized entities.
Remote to local attacks	Access to hosts is gained by unauthorized entities.

**Table 7:** Performance analysis.

Models	Accuracy (%)	Precision (%)	Recall (%)	F1-Score (%)
Random forest	92.36	92.36	93.15	94.56
Decision tree	93.25	91.26	93.25	95.62
Logistic regression	92.23	96.25	94.15	96.32
Naïve Bayes	89.65	90.47	91.25	92.25
Support vector machine	92.36	94.26	93.25	92.25
MLP	89.65	88.14	89.25	92.15
Proposed	98.58	97.68	98.59	99.01

**Table 8:** Comparative analysis.

Methods	Dataset	Accuracy (in %)
Proposed	Drone dataset	98.58
Proposed	NSL-KDD	98.69
Proposed	KDD CUP 99	99.01
PCA + MCA	Drone dataset	92.25
Deep neural network	KDD CUP 99	91.25
DT–RF	NSL-KDD	89.69
PCA + MCA	Drone dataset	92.58
Deep neural network	KDD CUP 99	93.25
DT–RF	NSL-KDD	91.25

## Data Availability

Not applicable.
